# Supplementary material for: Are carbon emissions trading and green financial instruments synergistic? -Comprehensive quantitative research based on content analysis
Source: PLoS One. 2024 Mar 7;19(3):e0298601. doi: 10.1371/journal.pone.0298601 (PMC10919601; doi:10.1371/journal.pone.0298601)
Supplement: S1 Data — (ZIP) [file pone.0298601.s001.zip › Data/地级市Z_F工作报告.zip!/╜¡╬≈/╕o╓▌╩╨/└┤╫╘╣1⁄2╓┌║┼í╛┬φ┐╦╩2╛▌═°í┐_2018.docx]

各位代表：

　　现在，我代表市人民政府向大会报告工作，请予审议，并请各位市政协委员和列席会议的同志提出意见。

　　一、2017年工作回顾

　　过去一年，在省委、省政府和市委的坚强领导下，全市上下以习近平新时代中国特色社会主义思想为指引，全面落实省委“创新引领、绿色崛起、担当实干、兴赣富民”工作方针，按照市委“一个坚持、三项重点、四个排头兵”的工作要求，坚持稳中求进工作总基调，牢固树立新发展理念，主动适应经济发展新常态，攻坚克难，奋勇拼搏，经济社会保持了稳中有进、进中向好的发展态势。2017年，全市实现生产总值1354.57亿元，增长8.7%；财政总收入183.64亿元，增长7%，其中一般公共预算收入120.19亿元，下降2.1%；规模以上工业增加值增长9.4%；固定资产投资1401.41亿元，增长12%；社会消费品零售总额537.66亿元，增长12%；出口总额129.48亿元，增长8.8%；实际利用外商直接投资3.53亿美元，增长9.1%；城镇居民人均可支配收入29463元，增长8.3%；农村居民人均可支配收入13563元，增长9%；城镇新增就业4.33万人；居民消费价格指数涨幅控制在2%以内。

　　一年来，我们主要做了以下几个方面工作：

　　——精准施策应对下行压力，经济保持平稳增长

　　充分发挥政策的放大效应。落实省降成本优环境130条和市配套出台的38条等政策措施，“深入”“精准”推进降成本优环境专项行动，累计为企业减负70.5亿元。出台了我市贯彻新理念培育新动能、深入实施工业强市战略、加快培育百亿企业、加快建设现代农业强市、发展总部经济、促进建筑业持续健康发展等系列政策措施，取得较好成效。设立12支总规模271亿元的市级产业引导基金。实现政府转贷机制县域全覆盖。严格商品房预售管控，房地产市场保持平稳健康发展。

　　充分发挥投资的关键作用。实施214个市重大重点项目，完成投资255亿元。41个项目列入省大中型项目，50个项目列入省市县三级联动项目。投资对GDP的贡献率达42.2%。资光、船广、东昌高速建成通车，“四横三纵一连”的高速路网格局初步显现。东外环高速、广昌至吉安高速、G316资溪花山界至南城黄狮渡段改建工程、G322宜黄黄陂至乐安鳌溪段新建工程等项目加快推进，“五纵七横十联”的国省道干线路网得到完善。抚州机场、吉抚武温铁路、昌抚城际铁路、南丰至瑞金城际铁路（鹰梅铁路先建段）、500千伏东乡输变电工程等重点项目的前期工作扎实开展。22个总投资189亿元的重大PPP项目全面推进，入选全国重大市政工程领域PPP创新工作重点城市。

　　充分发挥消费的基础作用。大力实施信息、养老、家政、健康等领域促进消费工程，消费对经济增长的贡献率达53.1%，首次超过投资对经济增长的贡献率。被列为全省唯一的中国电信全国八大云计算骨干节点地级市，在全省率先实现了全市光网全覆盖。中科慧康居家养老服务中心投入运营，亲和源大型养老社区建设快速推进。医养结合服务网络逐步形成，智慧百乡千村健康医养扶贫工程稳步推进，已在市中心城区和临川、南丰、广昌、崇仁四个县（区）开展试点。

　　——深化改革扩大开放，经济社会发展动力不断增强

　　改革步伐蹄疾步稳。放管服改革深入推进。市重点建设项目审批绿色通道和中介服务市场规范化建设取得重大成果，项目审批所需时间压缩70%以上。“多证合一”以及“互联网+二维码”的信用监管改革全面推开。推行“只跑一次”审批改革试点，梳理出15项政务服务事项，实行网上全流程办理。资溪县启动相对集中行政许可权改革试点，金溪、广昌、崇仁在全省先行开展城乡管理领域相对集中行政处罚权工作。乡镇便民服务中心全面建立，便民服务向村组延伸。农业农村体制改革稳步推进。全面完成农村土地确权登记验收工作，登记颁证率达97.6%。市农村综合产权交易中心建成投入使用，完成交易701宗。东乡区省级水权试点改革通过国家验收。公安“大部制”警务改革走在全国前列，全市刑事案件立案数大幅下降，街面“两抢”案件基本保持了零发案。出台了《市区财税体制改革的实施意见》，优化了市、区财税体制。与此同时，投融资体制、国资国企等重点领域改革取得重要进展。

　　区域合作步伐加快。全面落实昌抚“1+8”合作协议。两地共同签署了旅游区域合作框架协议，新增互送游客10万余名。完成昌抚合作示范区总规、核心区云山镇总规修编。投资36亿元的田园综合体开工建设，引进了投资5.3亿元的山东铭宸集团高标准设施农业项目，增设福银高速昌抚合作示范区（云山）互通获得批准。赣闽产业合作示范区建设全面启动。抚州检验检疫大楼竣工开业。抚州海西综合物流园建设进展顺利，口岸联检大楼完成主体验收，铁路专用线即将开工建设。向莆铁路铁海联运集装箱快速班列、南丰蜜桔中欧冷链班列正式开通，标志着抚州对外开放全面融入“一带一路”倡议。东临新区获批组建，启动了总规及产业规划编制，东外环高速等重大基础设施建设加快推进，抚州中心城区与东乡城区的对接开始加速。

　　对外开放持续扩大。招大引强成效明显。在北京、上海、深圳、香港等地举办16场新经济招商活动，签约项目177个，签约总额1450亿元。出口稳步回升。机电、高新产品、香精香料预计全年出口总额130亿元，增长9.4%。

　　——培育壮大发展新动能，产业结构加快升级

　　创新驱动发展战略深入实施。开展全社会研发投入专项行动，预计全社会R&D经费支出占GDP比重提高到0.8%以上。科技进步对经济增长贡献率达52.2%。全市高新技术企业135家，同比增长57%。深入开展“双返双创”活动，引进在外抚州籍企业家返乡投资2000万元以上项目120个，占全市落户招商引资总数42.6%。新聘2010年诺贝尔化学奖得主根岸英一以及9位中科院、工程院院士为市政府科技顾问，组建院士工作站10家。新增省级工程技术研究中心6家、国家知识产权优势企业6家，新增东乡区、金溪县、南丰县3个国家知识产权试点示范县（区），新增数量均列全省第一。获批国家知识产权试点城市。全市所有高新技术企业、60%以上规上工业企业、市直主要部门都与高等院校、科研机构建立科技合作关系。高新区生物医药产业纳入国家创新型产业集群试点。崇仁县工业园区创建“全国知名品牌创建示范区”进入国家复审。博雅生物荣获井冈质量奖提名奖，实现零的突破。

　　发展新动能逐步增强。新兴产业增加值同比增长15%。抚州高新区“两车一云一芯一药”快速发展，投资50亿元年产20万辆整车的大乘汽车科技产业园项目、投资50亿元的沃特玛新能源创新联盟建设新能源汽车项目、投资40亿元的超算中心项目、投资10亿元的半导体新材料产业园项目和投资35亿元的建昌帮药谷项目等重大产业项目加快推进。资溪大觉山成功创建国家5A级景区，实现我市5A级景区零的突破；南城麻姑山景区、金溪竹桥景区、宜黄曹山景区、广昌中国莲花景区、黎川古城景区通过国家4A级景区评审；文昌里历史文化街区快速推进，玉隆万寿宫修缮等6个项目全面完工；成功推出临川才子研学游和汤显祖、莎士比亚东西方文化之旅两条精品线路，开通旅游专列180余列，接待境外游客同比增加10倍。全市旅游接待总人数、旅游总收入分别增长35%和40%。入选2017中国文化竞争力十佳城市，全市文化产业主营业务收入、文化产业增加值分别增长17.2%和17.8%。“旴江医学”和“建昌药帮”老字号振兴步伐加快，建昌帮药业、广明实业等中医药龙头企业落户抚州，全市道地药材种植基地扩大到40万亩以上。存贷款余额突破3300亿元，贷款余额增速列全省第三。新增开业交通银行抚州分行，新增开业恒邦财险、渤海财险、信泰人寿3家保险公司。科技金融小镇一期工程开工建设，5家网络小额贷款公司开业运营，科技金融发展走在全省前列。新增南城博君农业在澳大利亚主板上市，新增3家企业在“新三板”挂牌。成功举办江西省第三届互联网大会，互联网产业专题招商对接会签约13个项目，投资额达77亿元。

　　传统产业改造升级提速。积极推进百亿企业培育工程，博雅欣和、迪比科新能源、金品铜科、森鸿科技、海利不锈钢、德义半导体、自立铜业等龙头企业加快成长。企业技改“三百计划”完成投资230亿元。智能化改造“千百十个”工程完成推广400台智能制造装备、21个智能制造车间、培育4家智能装备企业、创建1个智能制造园区的任务。新增高新区电子信息产业和黎川县日用耐热陶瓷产业2个省级重点产业集群，总数达到5个。中阳研砼装配式建筑生产基地正式开工。博雅欣和被列为国家绿色制造示范企业。博雅生物、明正变电等9家企业列入第一批省级军民融合企业。全市规模以上工业增加值增幅列全省第二，工业用电量增幅列全省第一。

　　现代农业提质增效。粮食生产实现“十四连丰”。出台《关于鼓励社会资本发展现代农业的指导意见》，制定16条针对性强、含金量高的扶持政策，引进重大现代农业项目20个，签约资金30亿元。率先在全省实现县（区）现代农业园区管委会全覆盖，全市现代农业示范区加快建设，入园项目实现产值162.65亿元。统筹整合资金推动34.7万亩高标准农田建设，引进社会资本发展1万亩高标准设施农业。南丰龟鳖、乐安稻鱼、黎川稻鸭等生态养殖模式效益显现。新增“三品一标”农产品认证93个，总数达530个。崇仁麻鸡被授予国家级农产品地理标志示范样板；南丰县成功创建南丰蜜桔中国特色农产品优势区；资溪县入选全县域有机农产品认证创建单位；南城县入选国家农产品质量安全监管县创建单位；南丰蜜桔栽培系统、广昌莲作文化系统被农业部认定为中国重要农业文化遗产。

　　——推进生态文明先行示范市建设，绿色发展优势持续提升

　　环境污染治理力度加大。“净空、净水、净土”专项行动深入开展。围绕治理“四尘三烟三气”环境污染，集中开展四个专项整治行动，PM10年均值64.5微克/立方米，PM2.5年均值47.04微克/立方米，环境空气优良天数居全省前列。集中开展中央环保督察问题整改，市中心城区和各县（区）一批污染治理项目加速推进。市中心城区垃圾焚烧发电厂主体工程即将封顶，崇仁、南丰区域性垃圾焚烧发电厂选址确定。打造“河长制”升级版。推进农村生活污水治理，实施了东乡北港河与乐安鳌溪河流域综合治理工程，水质状况明显改善。累计投入4305万元，在禁养区关闭或搬迁畜禽养殖场70家。推广了东乡区生活垃圾一体化市场改革模式，已有10个县（区）实行了城乡环卫一体化。投入4000万元，深入实施全市域封山育林，全市森林覆盖率达66.14%。资溪县入选首批全国生态文明建设示范县。

　　重大生态工程全面推进。抚河流域生态保护与综合治理一期、廖坊灌区二期、凤岗河生态化治理等示范工程全面推进，临川温泉生态文明样板区建设初见端倪。广昌抚河源湿地公园、廖坊湿地公园、凤岗河湿地公园纳入国家级湿地公园创建单位名单。黎川岩泉国家森林公园列入第三批国家森林氧吧榜单。

　　生态文明制度体系加快构建。严守生态保护红线，在全省率先实施国土空间规划分类考核，出台水资源生态补偿实施办法，积极开展乡镇生态综合执法试点和生态司法体制改革，率先上线运行“生态云”平台，率先开展碳普惠制试点。编制了自然资源资产负债表，实施领导干部自然资源资产离任审计试点。获批国家生态产品价值实现机制和全国首批流域水环境综合治理与可持续发展试点。

　　——统筹城乡一体化发展，城乡面貌显著改善

　　城市功能不断加强。城镇化率达到48.4%，较上年提高1.75个百分点。老城区六条城市大道、汝水生态水厂等11个重点项目全面完成，王安石大桥、中洲大桥、才子大桥、科技文化传媒综合体、市民中心、老年大学等重点项目全面开工。浙江开元五星级酒店加快建设，香港铜锣湾和省出版集团投资的两个五星级酒店落地，希尔顿欢朋酒店等四星级特色商务型酒店启动建设，万达商业综合体投入运营，中心城区商贸业更加繁荣。实施市中心城区老旧社区改造、农贸市场和市容环卫等基础设施建设提升三年行动计划，城市功能进一步增强。基本完成160万m2棚改征迁任务，利用腾空土地规划建设一批优质学校、社区医院、健康养老、游园绿地等公共服务设施。广昌驿前镇入选国家特色小镇，临川温泉镇、金溪香谷小镇入选省级特色小镇。

　　秀美乡村建设成效显著。按照“拆得彻底、清得干净、改得美观、管得规范”的要求，开展17条交通主干道沿线环境综合治理大行动，拆除破旧房、附属房、猪牛栏、临时建筑1120万m2，清理拆除养殖场、采石场、砖瓦场660家，恢复植被247.8万m2。同时注重做到“五个结合”，涌现出东乡虎圩乡徐家村、临川湖南乡坪山村、资溪乌石镇新月畲族村、南城徐家镇排头村、宜黄二都乡帘前村、南丰桑田镇榨源村、乐安戴坊镇排下新村等一批秀美乡村建设示范点。深入开展农村移风易俗，实现所有行政村红白理事会全覆盖。

　　——千方百计补齐民生短板，人民群众获得感明显增强

　　精准脱贫深入推进。针对部分地方脱贫质量不高、村级集体经济薄弱等问题，扎实开展脱贫攻坚“百日行动”，深入实施“五大工程”，贫困地区生产生活条件明显改善。全面建立“五条保障线”，建档立卡贫困户大病住院个人负担比例降至10%以下。光伏扶贫成效显著，完成装机总容量列全省第三。预计全市扶贫对象将由2016年底的7.6万人减少到4.4万人，91个贫困村退出，广昌县脱贫摘帽将接受国家考核评估。

　　社会保障更加有力。新增城镇就业4.33万人，转移农村劳动力6.4万人。各项社会保险参保人数均完成年度目标。实现城乡居民医保一体化管理，大病保险及重特大疾病的补偿标准进一步提高。在全省率先开通跨省异地就医即时结算暨医保银医一卡通系统。

　　社会事业蓬勃发展。高考成绩继续保持全省领先。成为全省首批县域义务教育基本均衡设区市。中小学教育质量综合评价实验成果被教育部总结推广。临川教育集团总投资12亿元的三所民办实验学校全面开工建设。崇仁师范学校成功升格为抚州幼儿师范高等专科学校，江西中医药高等专科学校升本规划建设全面启动，南昌大学抚州医学院独立办学列入省“十三五”高校设置规划。启动城市公立医院综合改革，取消了实行多年的“以药补医”机制，在医疗资源布局、三医联动、现代医院管理、人事薪酬制度改革等方面取得突破性进展。被评为全国创建幸福家庭活动示范市。全省标准最高、规模最大的市救灾求助中心建成投入使用。举办“孝满才乡”评选表彰活动，开展清明节文明祭扫，树立了文明风尚。市第五届运动会在东乡区成功举行。

　　社会治理持续强化。完善市、县、乡、村四级公共法律服务中心，建立矛盾调处工作机制及专业性、行业性调处委员会，有效化解常见性、多发性矛盾纠纷，调处成功率达98.9%。崇仁一户一位“法律明白人”工作试点获得省委肯定。安全生产态势总体良好，社会治安秩序持续向好，公众安全感不断提升。在全省率先开展车辆超限超载集中整治，综合排名列全省第一。

　　——加强政府自身建设，行政效能和服务水平有效提高

　　把学习贯彻党的十九大和习近平总书记重要讲话精神作为首要政治任务，深学笃用习近平新时代中国特色社会主义思想。严格落实政府系统全面从严治党主体责任，深入推进党风廉政建设和作风建设。扎实推进“两学一做”学习教育常态化制度化，认真贯彻中央八项规定精神。全面推进法治政府建设。加强行政监察和审计监督，严肃查处各类违纪违法案件。自觉接受市人大及其常委会的法律监督和工作监督、市政协的民主监督以及社会舆论监督。办理人大代表建议246件、政协提案298件，满意率和基本满意率均达98%以上。

　　国防动员和后备力量建设不断加强，国防教育、民兵基层、兵员征集、“双拥”共建、军民融合等工作取得新的成绩。同时，食品药品监管、司法行政、民族宗教、涉台事务、审计、烟草、统计调查、人民防空、防震减灾、气象、水文、保密、档案、地方志、妇女儿童、老龄、残联、科协、红十字会、驻外联络等工作都有了新的提升。

　　尤其令人振奋和自豪的是，我们成功举办汤显祖戏剧节暨国际戏剧交流月活动，把临川文化融入“一带一路”倡议和中国文化“走出去”战略。邀请10个“一带一路”沿线国家（地区）最具代表性的演出团体，以及中央芭蕾舞团、中国京剧院等7个国内知名院团，举办了23场交流演出。完成了一批室内和露天剧场建设项目，开设了地方戏曲传承班，打造中国戏都迈出新步伐。乡音版旴河高腔《临川四梦》首次走出国门，在新西兰成功演出。应邀赴英参加中英高级别人文交流机制第五次会议，与英方达成了一系列开创性成果，抚州成为中英文化交流合作的地方典范，临川文化在国际舞台上的知名度、影响力进一步提升。

　　需要如实向各位代表报告的是，在经济发展进入新常态的大背景下，受经济运行下行和省以下财税体制改革调整等因素的影响，2017年全市固定资产投资、一般公共预算收入以及城乡居民人均可支配收入等4项指标略低于年初计划目标。但从总体上看，经济发展的质量进一步提升，2017年经济社会发展的主要预期目标任务基本完成。

　　各位代表！过去一年取得的成绩来之不易。这是省委、省政府和市委坚强领导的结果，是全市干部群众开拓奋进、社会各界鼎力支持的结果。在此，我代表市人民政府，向全市人民，向各位人大代表和政协委员、各民主党派、工商联、无党派人士、各人民团体，向中央和省驻抚单位，向驻抚部队指战员、武警消防官兵、公安干警，向所有关心支持抚州改革发展稳定的同志们及各界人士，表示衷心的感谢和崇高的敬意！

　　我们也清醒地认识到，全市经济社会发展仍然存在不少困难和问题。主要是：发展不足问题仍然突出，经济总量偏小，产业层次不高，区域经济发展不平衡；创新能力仍显薄弱，传统产业转型升级不快，新动能培育不足，实体经济还有不少困难；就业、教育、医疗、养老、环保等民生领域短板较为突出；一些部门工作作风不扎实，服务质量和效率不高，少数干部不作为、不会为、不敢为现象依然存在等等。对此，我们一定会重视发展中的问题，正视工作中的差距，不遮掩、不回避、不懈怠，采取坚决有力措施切实加以解决。

　　二、2018年工作总体安排

　　2018年是贯彻党的十九大精神的开局之年，是改革开放40周年，是决胜全面建成小康社会、实施“十三五”规划承上启下的关键一年，做好全市经济社会发展工作至关重要、意义重大。我们既要看到经济下行压力仍然较大，更要看到我市发展面临的难得宝贵机遇，全市基础设施加快完善，生态优势持续提升，新产业、新业态方兴未艾，抚州人民的文化自觉、文化自信更加坚定，只要我们保持定力、尽锐出战、精准施策、持续发力，就一定能够把抚州的事情办得更好，向历史和人民交出一份满意答卷！

　　今年政府工作的总体要求是：以习近平新时代中国特色社会主义思想为指引，全面贯彻党的十九大、中央经济工作会议和省委十四届五次全会、市委四届四次全会精神，按照高质量发展的要求，坚持稳中求进工作总基调，坚持新发展理念，坚持以供给侧结构性改革为主线，围绕市委“坚持一个指导、突出三项攻坚、打好四大升级战”的工作要求，聚焦构建现代化经济体系，聚焦深化改革开放，聚焦推进生态文明先行示范市建设，聚焦打好脱贫攻坚战，聚焦保障和改善民生，促进经济社会持续健康发展，奋力迈出“科学发展、绿色崛起，全面建成小康新抚州”的新步伐，谱写新时代中国特色社会主义抚州篇章。

　　今年经济社会发展的主要预期目标是：生产总值增长8.8%；财政总收入增长9%，其中一般公共预算收入增长7%；规模以上工业增加值增长9%以上；固定资产投资增长12%；社会消费品零售总额增长12%；出口总额增长3%；实际利用外商直接投资增长10%；城镇居民人均可支配收入增长8.5%以上；农村居民人均可支配收入增长9%以上。

　　围绕上述目标，重点做好以下八个方面工作：

　　（一）深挖供需两端潜力，不断增强经济发展的内生动力

　　精准扩大有效投资。围绕基础设施、产业提升、公共服务等重点领域，充分发挥投资对优化供给结构的关键性作用。推进和实施256个全市重大重点项目建设，总投资1506亿元，力争今年完成投资566亿元以上。加快东外环高速、广吉高速、G316资溪花山界至南城黄狮渡段公路改建、G322宜黄黄陂至乐安鳌溪段新建、抚河流域生态保护及综合治理一期、廖坊灌区二期、临川温泉生态文明样板区、快速大容量公交（BRT）等重大项目建设，完成升级改造国省道200公里左右；新开工建设西外环高速、贵资高速、昌莆加密高速（南昌—崇仁—宜黄—南丰段）、抚河生态文化旅游示范公路、抚河流域生态保护及综合治理二期、广昌和乐安220千伏输变电工程；做好抚州机场、南丰至瑞金城际铁路（鹰梅铁路先建段）、昌抚城际铁路、吉抚武温铁路等项目前期工作，争取抚州机场开工建设。加大对生物医药、电子信息、新能源汽车、新材料等战略性新兴产业投资强度，加快形成发展新动力。加大对教育、医疗、卫生、养老、环保等公共服务领域的投资力度，不断增强供给侧服务能力。

　　优化消费有效供给。统筹城乡消费市场建设。加快中心城区尤其是各县城的城市商业综合体建设，推动传统零售转型发展。完善乡村互联网设施建设，重点打造南城、乐安国家级电子商务进农村综合示范县，全面推进农村电商加快发展。促进健康养老消费。全面放开养老服务市场，深入推进医养结合。支持临川云山慢城、南城麻姑山打造养生养老产业基地，建设一批康养产业综合体。鼓励和支持各县（区）加快建设医养结合示范基地，助推亲和源等一批品牌养老机构落户抚州，加快发展。鼓励绿色消费。加大新能源汽车推广力度，大力推广新能源公交车，发展新能源物流车、货运车，加快充电桩等配套基础设施建设。继续推广应用高效节能电机、LED照明等节能产品。严格执行政府对节能环保产品的优先采购和强制采购制度，扩大政府绿色采购范围。

　　大力振兴实体经济。深入持续推进降成本优环境专项行动。继续在深入和精准上下功夫，加大减税降费力度，加强园区功能性平台建设，精简园区企业审批事项，有效降低企业要素成本和制度性交易成本，切实减轻企业负担，着力解决金融帮扶“脱实向虚”问题。打好开发区改革和创新发展攻坚战。加快开发区转型升级。更加突出创新驱动发展、产业集聚发展、绿色集约发展，建立科学合理的考核评价体系，全力支持国家高新区改革创新发展。聚焦政策、资源、服务，支持高新区尽快打造1个过五百亿、2-3个以上过双百亿产业，尽快迈上千亿园区台阶。各县（区）重点打造1-2个以上双百亿产业，并做大做强。支持县（区）工业园区创建省级产业园区。完善开发区管理机制。深化开发区人事和薪酬制度改革，理顺开发区财税管理体制。加快开发区市场化改革，支持开发区引进国内外战略投资者、专业化园区运营商，以“整体外包”“特许经营”等形式，开展运营合作。完善功能性配套设施，大力提升硬环境；完善综合服务管理体系，建立健全常态化的政企沟通机制、问题协调处理机制和投诉问责机制，着力优化软环境，打造高水平营商环境。激活和扩大民间投资。加大基础设施和公用事业领域向民间资本开放力度，依法维护民营企业合法权益，为民营企业创造平等竞争机会。全面实施市场准入负面清单制度，落实公平竞争审查制度，激发各类市场主体活力。激发和保护企业家精神，构建“亲”“清”新型政商关系，营造尊重和激励企业家干事创业的社会氛围。

　　（二）推进建设创新型城市，加快发展动能接续转换

　　提升创新引领能力。深入开展“加大全社会研发投入攻坚行动”。加大政府科技投入，发挥财政对企业、高校、科研机构等全社会科技投入的引领激励作用，最大强度地聚集更多的社会资本投入科技创新领域，力争R&D经费支出占GDP的比重提高到1.25%。全面建立市、县政府科技顾问制度，市直部门科技支撑制度，实现规上企业科技合作全覆盖。提升自主创新能力。强化企业创新主体地位，抓好质量兴市战略，形成一批拥有核心竞争力的实体企业。全面推进企业与高校、科研机构科技合作，加强院士工作站建设，加快企业技术中心、工程技术研究中心、重点实验室等国家和省级企业创新平台建设。聚焦新兴产业和优势产业，力争在电子信息、生物医药、智能装备、新能源、新材料等领域突破一批关键共性技术，抢占产业发展制高点。深化科技体制改革。高度重视招才引智，积极推进人才强市战略，落实《抚州市高层次人才引进暂行办法》等一揽子人才新政策。深入推进“双返双创”活动，突出引进基础性人才、高技能人才、青年科技人才和高水平创新团队。支持抚州高新区创建鄱阳湖国家自主创新示范区。支持科技型中小企业开展科技成果转化，强化知识产权保护。积极创建国家知识产权示范城市。

　　培育壮大新兴产业。围绕休闲旅游、文化创意、健康养老、中医药、现代物流、数字经济等六大新兴产业，以产业园区、特色小镇为主要载体，建立集聚集群集约的新兴产业体系。以举办好2018年全省旅游发展大会为契机，推动文化和旅游深度融合，争创全国全域旅游示范市。大力发展古村、民宿等乡村体验游，力争创建1家省5A、10家以上省4A乡村旅游点。规划建设1-2个自驾车房车露营地，争取三翁花园、抚州一中校区、临川灵谷峰、东乡佛岭公园、南丰潭湖、资溪御龙湾、乐安金竹瀑布、崇仁山凤景区创建国家4A级景区，确保实现“县县都有重点旅游景区”目标。争创1家五星级酒店（浙江开元）、4家四星级酒店。围绕振兴“旴江医学”“建昌帮”，大力发展道地中药材种植，力争每个县（区）建立1-3个万亩以上道地中药材种植基地。推动线上线下中药材交易中心以及中药材检测检验中心建设，以建昌帮药业、南城广明实业、银涛药业等重点企业为龙头，着力打造中医药强市。以建设物流大通道、完善物流基础设施为重点，积极引导推动一批物流企业股改上市，推动物流产业信息化、标准化、集约化发展，全力打造区域性现代物流中心。加快推进曙光政务云计算中心、卓朗大数据中心、创世纪超算中心项目建设，全力打造全省大数据重点产业基地。推动军民融合发展，培育更多的军民融合优势企业。

　　推动传统产业转型升级。围绕壮大机电汽车、生物医药、电子信息、建筑建材、食品加工和有色金属深加工等六大主导产业，深入实施“百亿企业”培育工程，继续实施重大技改“三百计划”、智能制造“千百十个”工程，力争全年技改投资占投资比重达25%以上。推动信息化与工业化深度融合，加快云计算、物联网、传感控制、机器人等技术应用，推动传统产业迈向中高端。加快推进大乘汽车、沃特玛、迪比科等新能源汽车项目建设，着力引进一批电池、电机、电控骨干企业，形成全产业链条，打造全省新能源汽车生产基地。大力发展森鸿、赛华、联益等一批电子信息企业，打造1-2个具有较强竞争力的新型电子产业基地。抓好金品铜科、海利不锈钢、志特新材料、德义半导体等企业发展，打造全国重要的新材料研发生产基地。重点支持抚州高新区汽车及零部件、电子信息、生物医药，崇仁变电设备，金溪香精香料，黎川日用耐热陶瓷等省级产业集群做大做强，力争全市百亿元以上产业集群突破3个，重点产业集群主营业务收入尽快突破500亿元。加快推进中阳研砼、玉茗远大装配式建筑生产基地建设，在政府投资项目中加快实施一批装配式建筑示范项目，形成装配式建筑产业集群。大力弘扬工匠精神，引导企业树立质量为先、信誉至上的经营理念，创造“抚州制造”的辉煌。

　　（三）强化改革攻坚力度，厚植经济发展优势

　　突出抓好供给侧结构性改革。坚决淘汰落后产能，用市场化法治化方式去产能，依法依规处置“僵尸企业”。坚持“房子是用来住的，不是用来炒的”定位，加大廉租房、公租房、拆迁安置房、“三限房”、人才公寓的建设力度，增加土地市场的供应量，规范商品房销售管理，大力培育住房租赁市场，优化住房供给结构，促进房地产健康平稳发展。坚持金融服务实体经济的本质要求，加大信贷投放力度，切实增加对实体经济的资本供给。充分运用股权融资、市场化债转股等多种手段，积极稳妥降低企业杠杆率。健全金融征信体系，完善金融风险监测预警机制，依法严厉打击非法集资等违法金融活动，坚决守住不发生系统性区域性金融风险的底线。

　　加快推进重点领域改革。深化放管服改革。持续深化简政放权，再取消或下放一批制约企业生产、投资经营活动和群众办事不便的行政权力事项。全面开放中介服务市场，加强中介超市管理。切实增强监管能力，确保实现全市“双随机一公开”监管全覆盖。积极探索对新技术、新产业、新业态、新模式包容创新的审慎监管制度，为新动能成长打开更大空间。大力提升服务水平，加快推进“互联网+政务服务”，全面推行“最多跑一次”和“一次不跑”改革，真正实现“数据多跑路，群众少跑腿”。深化国资国企改革。落实国有资产保值增值责任，全面推行经营性国有资产与主管单位脱钩，建立权责一致的市属经营性国有资产监管全覆盖体系，完善投资运营公司实体化转型改革，完善公司法人治理结构和市场化经营机制。推进国有企业混合所有制改革，抓好江铃轻汽混改试点工作。深化财税体制改革。完善地方政府债务管理，用好用足地方政府债券资金，防范化解财政风险。继续深化政府采购制度改革。稳步推进事业单位公车改革。深化金融和投融资体制改革。大力发展科技金融，推动设立全省科技金融创新试验区，吸引更多合规科技金融机构落户抚州。丰富现代金融业态，力争在引进股份制商业银行、融资租赁公司等机构上取得突破。发挥各级政府产业基金的引导作用，支持符合条件的县（区）和企业发行各类债券，股债并推提升直接融资比重。用足用好贫困县IPO绿色通道政策，力争广昌志特新材料和东乡施美制药2家企业在创业板上市，力推更多企业到“新三板”和区域性股权市场挂牌融资。深化公安“大部制”警务改革。进一步推动公安机关合并、职能整合等改革创新，提升警务现代化能力和水平，打造全国公安机关可复制、可推广的“大部制”警务改革“抚州样板”。

　　（四）全面扩大对外开放，增强区域发展活力

　　大力发展开放型经济。全力打造开放平台。努力构建连通赣闽的物流大通道，加快推进抚州海西综合物流园、国家应急储备中心、邮件快件处理中心、江西移动物流中心等产业平台建设，争取赣闽合作陆地港尽快落地，推进向莆铁路铁海联运货运班列、中欧货运集装箱班列常态化运行。推动口岸平台建设，尽快设立抚州海关，争取设立崇仁、金溪国检监管试验区，加强国际贸易“单一窗口”国家标准版的推广应用，打造国际化、便利化的营商环境。加快外贸发展方式转变。大力支持和鼓励自营出口型实体企业发展，提高出口质量和效益。加快培育一批出口超千万、超五千万和超亿美元的重点生产出口企业。鼓励有条件的企业跨国（境）发展。开展精准招商。探索试行委托招商、代理招商，积极打造高水平专业化招商团队，围绕产业鱼骨图开展产业链上下游强链、补链、壮链招商。市、县（区）围绕重点产业，共同举办专题招商活动，提高招商精准化水平。推进“央企入抚、外企入抚、民企入抚、赣商返抚”四大工程，突出招大引强，重点瞄准世界500强、国内500强、行业前10强、主板上市公司，力争全年引进10个以上重特大产业龙头项目。

　　加快推动区域合作。加速推进昌抚一体化，全面融入南昌大都市区。全面落实“1+8”合作协议，加强重大政策和重要事项的沟通协商。着力推进昌抚合作示范区建设，聚焦发展旅游休闲、教育文化、养生养老、绿色农业等特色产业，努力将其打造成为昌抚合作的重要支点、新型城镇化的示范基地、展示抚州新形象的示范区域。加快建设向莆经济带，全面对接海西经济区。用足用好《关于支持抚州依托向莆经济带进一步开放开发的政策措施》，全方位对接融入国家战略，加强与“一带一路”沿线国家的经贸合作，加快打造成为联接“海上丝绸之路”与“陆上丝绸之路”的重要节点城市。加快推进赣闽产业合作示范区建设，努力将其打造成赣闽产业合作引爆点、省级合作体制机制创新区和区域发展升级新引擎。加快原中央苏区振兴步伐，争取和用好中央支持政策。积极谋划争取国家支持的政策措施，加大对我市重大基础设施、特色优势产业升级、改革创新先行先试的支持力度。

　　深入推进新型城镇化。强力推动“城市双修”。深入推进以人为核心的城镇化，抓好一批“城市双修”重点建设项目。修复城市生态。全面落实海绵城市建设理念，综合整治城市黑臭水体，对凤岗河、梦湖全面实施控源截污，增强水体自净能力；完善绿地系统，通过拆旧建绿、破硬复绿、见缝插绿等方式，拓展绿色空间，构建完整连贯的城市绿地系统。修补城市功能。加快老旧管网改造，大力完善城市给水、排水、燃气、通信、电力等基础设施，加快地下综合管廊建设；改善出行条件，全面打通城市丁字路、断头路，形成完整路网体系；新建及改扩建一批地下停车场、立体停车楼，增加城市停车位；新改扩建一批农贸市场，持续推进市中心城区老旧社区改造三年行动计划；开展市容大整治，重点提升城郊和城中村环境，实施东南西北四个城市出入口提升改造工程；推行城市管理市场化改革，加强城市精细化管理，加快完善“天上有云、地上有格、格中有人、人人有责”的数字城管建设。推进城乡融合发展。加快农业转移人口市民化，深化户籍制度改革，全面放开农业转移人口落户条件，完善进城农民基本公共服务体系。坚持以工补农、以城带乡，加快公共服务向农村覆盖，推动农村基础设施建设提档升级，逐步实现城乡公共资源配置适度均衡和基本公共服务均等化。探索城市组团式发展。推进市中心城区与东乡、南城、金溪、崇仁等周边县（区）同城化、组团式发展。构建便捷的区域交通体系，尽快启动市中心城区至周边县（区）快速通道建设，开通抚州至南城、抚州至宜黄城际公交。大力发展飞地经济，加快东临新区规划编制，尽快启动基础设施建设，努力将东临新区打造为抚州高新技术企业聚集区、战略性新兴产业样板区、生态文明和经济发展融合区。

　　（五）大力实施乡村振兴战略，加快推进农业农村现代化

　　深化农业农村改革。积极推进农村土地“三权分置”改革，在落实集体所有权、保障农民承包权的前提下，以放活土地经营权为重点，促进土地经营权有序流转，推动农业适度规模经营，大力发展现代农业。巩固农村土地承包经营权确权登记颁证工作成果，认真落实好第二轮土地承包到期后再延长三十年的政策。推进农村集体产权制度改革，盘活农村资产资源，保障农民财产权益，培育壮大村级集体经济，到2020年基本消除“空壳村”。

　　加快现代农业发展步伐。推进现代农业示范园区建设。促进要素集中、产业集群、政策集成，加快一二三产业融合发展，形成龙头企业引领、新兴经营主体为主、小农户参与的现代农业“雁阵”格局。大力开展农业招商，加快推进高标准农田、设施农业建设，做好土地开发和耕地占补平衡工作，建成高标准绿色生态农田30万亩以上。优化农业产业结构。积极落实粮食安全省长负责制，在稳定粮食综合生产能力的基础上，加快农业产业结构调整，大力实施九大农业发展工程，重点发展“两特一游”（即以中药材、生态水果、出口蔬菜为重点的特色种植，以生态禽畜、特色水产为重点的特色养殖，发展乡村休闲旅游），力争粮食产量稳定在55亿斤左右，优质农产品结构调整规模达到20万亩以上。坚持质量兴农、绿色兴农，加快推进农业绿色品牌建设，新增具有区域优势特色和国际国内竞争力的地理标志商标2个，新增无公害农产品、绿色食品、有机农产品品牌30个以上。

　　推进美丽乡村建设。坚持连线成片和贫困村优先原则，继续选择2700个左右村点开展秀美乡村建设，突出抓好村庄整治建设，补齐基础设施和公共服务短板，全面改善农民生产生活条件。积极开展美丽示范“六统四联创”活动，打造一批美丽示范标杆。全面完成包括辖区内铁路、公路沿线在内的农村环境综合整治。积极探索以“第三方治理”市场化为主要形式的长效管理机制，确保上半年通过国家农村垃圾专项治理达标验收。

　　完善乡村治理体系。深化农村精神文明建设，推进农村移风易俗，健全自治、法治、德治相结合的乡村治理体系。积极稳妥推进村（社区）“两委”换届选举工作，完善民主管理和基层自治。发展职业农民队伍，培养造就一支懂农业、爱农村、爱农民的基层“三农”工作队伍。

　　（六）着力提升生态环境质量，打造生态文明建设“抚州样板”

　　持续推进生态修复治理工程。坚持山水林田湖草生命共同体理念，基本完成抚河流域生态保护及综合治理一期工程、廖坊水利枢纽灌区二期主体工程。加快推进临川温泉生态文明样板区建设。开工建设抚河流域生态保护及综合治理二期工程，推进抚河生态文化旅游示范公路建设，争取山水林田湖草综合治理项目列入国家试点，力争重大生态工程建设取得明显进展。

　　着力解决突出环境问题。深入实施“净空、净水、净土”行动。打好蓝天保卫战，建立大气污染预警预报系统，加强重点大气污染源防控，加强市中心城区烟花爆竹燃放限禁管理，持续推进四个专项行动，确保空气质量优良天数占比继续位居全省前列。抓好国家级流域水环境综合治理与可持续发展试点。深入实施“河长制”升级版，加大水库、湖泊天然放养管理力度，建立以流域为单元的水环境综合治理体系，加快推进凤岗河、梦湖、东乡北港河、乐安鳌溪河和崇仁、宜黄小流域治理工程。落实土壤污染风险管控机制，加大农业面源污染防治力度，加强农村生活污水、生活垃圾处理。开展市中心城区生活垃圾分类处理试点工作，推行餐厨垃圾无害化处理。加快市中心城区垃圾焚烧发电厂建设，确保6月份投入使用。动工兴建南丰、崇仁区域性垃圾焚烧发电项目。深入推进城乡“厕所革命”三年行动计划，加快补齐影响群众生活品质的短板。倡导绿色生活方式，开展碳普惠制试点，建设绿色低碳城市。

　　巩固提升绿色发展水平。推进生态循环农业发展。实施化肥农药使用量零增长行动，推广有机肥替代化肥、测土配方施肥，强化病虫害统防统治和全程绿色防控。严格依法落实秸秆禁烧制度，推广先进的农机设备和作业标准，优先开展就地还田，推进秸秆全量化综合利用。推进绿色低碳工业发展。大力推进绿色工厂建设，推动企业循环式生产、产业循环式组合、园区循环化改造，培育节能减排科技创新示范企业。加快资源共享、废物处理、服务高效的公共平台建设，促进废物交换利用、能量梯级利用、水的分类利用和循环使用。推进集约高效服务业发展。提高生活性服务业品质，促进生产性服务业专业化发展。加强物流标准化建设，推进铁路、公路、水路、民航等多式联运发展。支持发展工业设计、计量测试、标准试验验证、检验检测认证等高技术服务业。

　　健全生态文明制度体系。完善主体功能区考核机制，推动生态保护补偿逐步覆盖到森林、湿地、耕地等重点领域。全面开展生态文明综合评价考核，编制自然资源资产负债表，建立完善领导干部自然资源资产离任审计制度。创新生态文明市场化机制，建立市级综合性生态资源交易平台。加快推进环保机构监测执法垂直管理改革，推广生态环境综合执法和城乡管理领域相对集中行政处罚权执法。重点开展国家生态产品价值实现机制试点和流域水环境综合治理与可持续发展试点，力争形成“绿水青山就是金山银山”的实践样板。

　　（七）充分发挥文化优势，加快建设文化强市

　　加强思想道德建设。坚持用社会主义核心价值观凝魂聚力，深入开展群众性精神文明创建活动，推动诚信建设和学雷锋志愿服务制度化。市中心城区启动国家文明城市创建，支持符合条件的县（区）创建国家、省级文明城市。

　　推动文化事业与产业发展。完善公共文化服务体系，大力实施全民阅读、农村电影放映等文化惠民工程。致力把“汤显祖”品牌转化为旅游发展优势，精心策划举办好首届汤显祖国际戏剧节。加快组建汤显祖艺术基金会，用市场手段推动汤显祖文化走出去。推动创办汤显祖艺术传媒职业学院，为全市的戏曲文化传承和文化产业发展培育人才。加快建设三翁戏曲小镇，打造文昌里国家级历史文化街区。设立文化产业发展基金，重点支持文化企业产业化市场化发展。加快文化创意产业园区建设，优化黎川油画、东乡木雕、高新区数字出版等文化产业布局。繁荣演艺市场，降低社会资本进入门槛，支持民营文化院团发展，推动汤显祖大剧院加入知名演艺院线。

　　加快历史文化名城创建。推进文化遗产保护利用工程，健全历史风貌保护，加快成片、成街坊、成区域保护。完成文昌里历史文化街区改造一期工程建设，全面推进二期工程。大力推进荆公路、州学岭历史文化街区改造和建设。鼓励老建筑改造再利用，优先将老厂区、旧厂房用于公共文化、公共体育、文化创意产业。加快市中心城区省级历史文化名城创建，启动国家历史文化名城申报工作，支持南丰、金溪创建省级历史文化名城。

　　（八）持续推进民生改善，不断满足人民群众新期待

　　坚决打赢精准脱贫攻坚战。按照“核心是精准、关键在落实、确保可持续”的要求，深入推进脱贫攻坚“五大工程”，加快实施一批农村道路交通、饮水安全、电网升级和危房改造项目，切实改善贫困地区生产生活条件。围绕“两不愁、三保障”脱贫标准，聚焦深度贫困村和特殊贫困人口精准帮扶，确保全年2.5万贫困人口脱贫、68个贫困村退出、国定贫困县乐安摘帽。巩固广昌县脱贫成果。同步推进城镇贫困和困难群体精准帮扶、脱贫解困工作。

　　尽心尽力办好民生实事。积极提高就业质量。坚持就业优先战略和积极就业政策，统筹做好高校毕业生、城镇就业困难人员、退役军人等群体就业工作，力争全年城镇新增就业3.67万人，新增转移农村劳动力5.52万人。加快建成多层次社会保障体系。加快推进“多险合一”系统建设，全面实施全民参保计划，建立统一的社会保险公共服务平台。加大棚户区改造力度。在全市实施59个棚户区改造项目，改造棚户区面积241万m2，其中市中心城区80.5万m2。优先发展教育事业。加快义务教育优质均衡发展，统筹推进县域内城乡义务教育一体化，逐步消除大班额、超大班额，切实化解“农村弱、城镇挤”的矛盾。普及学前教育，加快乡镇中心幼儿园等普惠性幼儿园建设，力争实现“一乡一园”目标。加强高中教育，加快临川教育集团3所实验学校、抚州市示范性综合实践基地、抚州市第二实验学校等教育重点工程建设。抓好教育信息化建设，更加关注教育公平，提升残健融合办学水平，保障特殊群体接受教育权利。发展职业教育，抓好抚州职业技术学院办学提升和抚州技师学院创办工作，推行校企合作，开展订单教育。提升高等教育，抓好江西中医药高专升本、抚州医学院独立办学和抚州幼专办学提升。加快健康抚州建设。深化医疗卫生体制改革，努力为人民群众提供全方位全周期健康服务。全面启动多种形式的医疗联合体建设试点，推动构建分级诊疗制度。支持市人民医院（市第一人民医院）做强做优，加快抚州市立医院（原抚州地区人民医院）独立建院、市中医院新建、市妇女儿童医院等卫生重点项目建设进度。全力构建“30分钟乡村医疗服务圈”，全面推进智慧百乡千村健康医养扶贫工程，加强基层医疗卫生服务体系和全科医生队伍建设，推进县福利院和乡镇“两院”达标提升融合工程。大力推进医养结合，市级建设一所三级康复医院和一所护理院，每个县（区）建设一所二级以上康复医院。加快市中心城区国家卫生城市创建步伐，支持东乡区创建国家卫生县城。实施食品安全战略，全面推进食品安全示范城市创建。推动体育事业发展。着力构建城市社区15分钟健身圈，鼓励和引导社会力量兴办全民健身事业。推进体育场馆管理体制变革，完善大型体育场馆免费或低收费开放政策，推动公共体育场地设施和符合开放条件的企事业单位、学校体育场地设施向社会开放。

　　打造共建共治共享的社会治理格局。着力加强社区治理体系建设，发挥好社会组织作用，推动基层治理触角向每个社区网格延伸，实现政府治理与社会调节、居民自治良性互动。全面推广崇仁一户一位“法律明白人”工作经验。畅通群众诉求表达与反应机制，加强行政复议、行政调解和人民调解工作，加快公共法律服务平台建设，完善法律援助制度。推进平安抚州建设，健全社会治安防控体系，依法坚决打击各类违法犯罪活动。坚决堵住安全生产漏洞，坚决防范重特大事故，坚决守住安全生产底线。落实信访工作责任制，建立健全社会风险评估机制，把社会稳定风险评估和信访风险评估、预警制度作为重大政策、重大改革、重大项目的前置程序和刚性门槛。

　　加强国防动员和后备力量建设，深化军民融合，做好人民防空和拥军优抚退役安置工作。

　　各位代表！面对新时代新使命新任务，全市政府系统要更加重视自身建设，切实加强学习本领、政治领导本领、改革创新本领、科学发展本领、依法执政本领、群众工作本领、狠抓落实本领和驾驭风险本领，增强政府执行力和公信力，切实提高群众的获得感、幸福感、安全感。一是旗帜鲜明讲政治。牢固树立“四个意识”，坚决维护以习近平同志为核心的党中央权威和集中统一领导，严格遵守政治纪律和政治规矩，始终在政治立场、政治方向、政治原则、政治道路上同党中央保持高度一致，确保政令通畅、令行禁止。二是坚持不懈强能力。围绕学习型政府建设要求，加强干部专业素养、专业能力、专业精神培养，增强理解力、执行力和操作力，努力建设一支高素质专业化的干部队伍，不断适应新时代中国特色社会主义发展要求。三是驰而不息改作风。深入推进“两学一做”学习教育常态化制度化，扎实开展“不忘初心、牢记使命”主题教育。认真贯彻落实习近平总书记关于进一步纠正“四风”、加强作风建设的重要指示精神，以更高标准、更严要求，驰而不息纠“四风”、改作风，推动作风建设向纵深发展。四是勇于负责敢担当。始终保持昂扬向上、奋发有为的精神状态，面对急难险重任务勇于负责任、挑重担，面对改革创新任务勇于啃“硬骨头”“涉险滩”。弘扬担当精神，为愿干事、敢干事、能干事、干成事的人提供施展才华的舞台，确保设定的目标、出台的政策、部署的任务，落到实处、见到实效。五是始终不渝抓廉洁。认真落实政府系统全面从严治党主体责任，切实履行“一岗双责”。严格遵守中央八项规定实施细则，开展扶贫领域腐败和作风问题专项治理，严厉惩治群众身边的“微腐败”，维护清正廉洁的政府形象。与此同时，加快法治政府建设，把政府工作全面纳入法治轨道，落实重大决策程序听证、风险评估和责任追究机制。认真执行人大及其常委会决议决定，主动向人大及其常委会报告工作，加强重大问题与政协的民主协商，自觉接受法律监督、工作监督、民主监督。认真办理人大代表建议和政协提案，切实提高建议和提案的办理质量。

　　各位代表！时代赋予重任，奋斗成就伟业。让我们更加紧密地团结在以习近平同志为核心的党中央周围,在省委、省政府和市委的坚强领导下,攻坚克难，真抓实干,奋发进取，砥砺前行，为全面建成小康新抚州、奋力谱写新时代抚州发展新篇章而不懈奋斗！
